# Carbon-Supported Mo_2_C for Oxygen Reduction Reaction Electrocatalysis

**DOI:** 10.3390/nano10091805

**Published:** 2020-09-10

**Authors:** Dušan Mladenović, Milica Vujković, Slavko Mentus, Diogo M. F. Santos, Raquel P. Rocha, Cesar A. C. Sequeira, Jose Luis Figueiredo, Biljana Šljukić

**Affiliations:** 1 Faculty of Physical Chemistry, University of Belgrade, Studentski trg 12-16, 11158 Belgrade, Serbia; dusan.mladenovic@ffh.bg.ac.rs (D.M.); milica.vujkovic@ffh.bg.ac.rs (M.V.); slavko@ffh.bg.ac.rs (S.M.); 2 Serbian Academy of Sciences and Arts, Kneza Mihaila 35, 11000 Belgrade, Serbia; 3 CeFEMA, Instituto Superior Técnico, Universidade de Lisboa, 1049-001 Lisbon, Portugal; diogosantos@tecnico.ulisboa.pt (D.M.F.S.); cesarsequeira@ist.utl.pt (C.A.C.S.); 4 Laboratory of Separation and Reaction Engineering-Laboratory of Catalysis and Materials (LSRE-LCM), Department of Chemical Engineering, Faculty of Engineering, University of Porto, Rua Dr. Roberto Frias, 4200-465 Porto, Portugal; rprocha@fe.up.pt (R.P.R.); jlfig@fe.up.pt (J.L.F.)

**Keywords:** oxygen reduction reaction, molybdenum carbide, carbon nanotubes, carbon xerogel, alkaline fuel cell

## Abstract

Molybdenum carbide (Mo_2_C)-based electrocatalysts were prepared using two different carbon supports, commercial carbon nanotubes (CNTs) and synthesised carbon xerogel (CXG), to be studied from the point of view of both capacitive and electrocatalytic properties. Cation type (K^+^ or Na^+^) in the alkaline electrolyte solution did not affect the rate of formation of the electrical double layer at a low scan rate of 10 mV s^−1^. Conversely, the different mobility of these cations through the electrolyte was found to be crucial for the rate of double-layer formation at higher scan rates. Molybdenum carbide supported on carbon xerogel (Mo_2_C/CXG) showed ca. 3 times higher double-layer capacity amounting to 75 mF cm^−2^ compared to molybdenum carbide supported on carbon nanotubes (Mo_2_C/CNT) with a value of 23 mF cm^−2^ due to having more than double the surface area size. The electrocatalytic properties of carbon-supported molybdenum carbides for the oxygen reduction reaction in alkaline media were evaluated using linear scan voltammetry with a rotating disk electrode. The studied materials demonstrated good electrocatalytic performance with Mo_2_C/CXG delivering higher current densities at more positive onset and half-wave potential. The number of electrons exchanged during oxygen reduction reaction (ORR) was calculated to be 3, suggesting a combination of four- and two-electron mechanism.

## 1. Introduction

Demand for sustainable and efficient energy sources to complement and eventually substitute existing fossil fuel-based ones has arisen with the increasing energy demand and environmental pollution. Electrochemical energy conversion devices such as fuel cells (FCs), batteries, and supercapacitors, are believed to be the most feasible alternatives among different energy technologies considered. Thus, the oxygen reduction reaction (ORR) has received much attention due to its application in fuel cells and metal-air batteries [1,2,3,4]. Sluggish ORR kinetics is one of the main limiting factors in the energy conversion efficiency of fuel cells and metal-air batteries, since ORR requires high overpotential and has a complex mechanism involving numerous steps [5,6,7,8]. ORR in alkaline media such as in alkaline fuel cells (AFCs) has faster kinetics, enabling the use of non-platinum (Pt) electrocatalysts in these cells [9]. However, Pt-based electrocatalysts are currently the most common electrocatalysts in AFCs and FCs in general, holding back their large-scale production and application.

Transition metal carbides (TMCs) are a potential alternative to replace Pt-based electrocatalysts due to the TMCs’ similar electronic density as Pt [10]. Furthermore, TMCs display high electrical conductivity, high oxidation resistance, high hardness, and high melting point. TMCs, such as molybdenum carbide (Mo_2_C), vanadium carbide (V_8_C_7_), and tungsten carbide (WC), have shown similar catalytic and electrocatalytic activities as Pt-group noble metals [11,12,13]. For instance, TMCs have been suggested as supports for Pt and Pt-group metals in fuel cells [14,15,16]. Pt-group metals supported on vanadium carbide (VC) and tantalum carbide (TaC) have been shown to have synergistic effects for the ORR, while zirconium carbide (ZrC) has shown to be a good support material for hydrogen oxidation reaction electrocatalysts [17,18,19]. Recently, WC, carbon-supported and unsupported, has been explored as an inexpensive, Pt-free electrocatalyst for ORR [10,20,21,22]. Carbon-supported Mo_2_C has also been reported to show high electrocatalytic activity for hydrogen evolution reaction (HER) [23,24], as well as for ORR [25,26,27,28]. TMCs’ advantage for applications in fuel cells is their higher abundance (they are several orders of magnitude more abundant than Pt) [29] and, consequently, lower price as electrocatalysts account for ca. 50% of a commercial fuel cell price.

TMC particles tend to agglomerate during their synthesis, especially under high-temperature reaction conditions [10]. However, large carbide particles display lower electrocatalytic activity compared to their nanosized counterparts due to too high densities and low specific surface areas. Thus, it is of great importance to develop nanosized TMCs with controllable particle size [14]. Further increase of electrocatalysts efficiency in FCs and metal-air batteries can be achieved by using high-surface-area and high-porosity support materials [30]. The support material should additionally be characterised by high electrical conductivity along with high corrosion-stability in different electrolytes used in fuel cells. Various types of carbon materials including carbon black, activated carbon, carbon nanotubes (CNTs), carbon xerogel (CXG), and graphene have been considered and tested as electrocatalyst supports for electrochemical energy conversion and storage devices [9,31,32,33]. CNTs and CXG have an advantage in terms of their 3D interconnected uniform pore structure that allows a high-degree dispersion of the active material and efficient diffusion of electrolyte [34]. Hence, electrocatalysts supported on CXG and CNTs have been reported to exhibit improved performance in comparison with those supported on conventional supports, such as Vulcan carbon black [35,36,37,38,39].

In our recent work, we have successfully prepared Mo_2_C supported on commercial CNTs (Mo_2_C/CNT) and synthesised CXG (Mo_2_C/CXG). In this work, Mo_2_C/CNT and Mo_2_C/CXG were tested as electrocatalysts for the ORR in alkaline fuel cells and for supercapacitors.

## 2. Materials and Methods 

### 2.1. Materials Preparation and Characterisation

The precursor of carbon xerogel (CXG) was prepared by polycondensation of resorcinol (Sigma-Aldrich, Taufkirchen, Germany) with formaldehyde (Sigma-Aldrich, Taufkirchen, Germany). After pH adjustment to produce a mesoporous structure, gelation was completed after 3 days at 85 °C in a paraffin bath. The sample was then dried (4 days in an oven at a temperature of 60–120 °C, increasing temperature by 20 °C per day) and carbonisation was performed by heating the material to 150, 400, 600, and 800 °C. Each temperature was kept for 1–6 h to result in CXG. The detailed procedure is described in previous work [24,40]. Multi-walled CNTs (NANOCYL, NC3100 series, Nanocyl, Sambreville, Belgium) were pre-treated with hydrochloric acid (HCl) (Sigma-Aldrich, Taufkirchen, Germany) [24]. The Mo_2_C electrocatalyst (Sigma-Aldrich, Taufkirchen, Germany) was supported on CXG and CNTs following a modified incipient wetness impregnation method [23,24]. Namely, an aqueous solution of ammonium molybdate (0.788 g in 6 mL, Sigma-Aldrich, Taufkirchen, Germany) was added dropwise to the carbon support (1 g), the slurry was homogenised in an ultrasonic bath (Model 3000514, JP Selecta, Barcelona, Spain) and dried at 110 °C overnight. Carburisation was performed in a tube furnace (Termolab, Águeda, Portugal) at a slow temperature rise to 800 °C and then holding it at 800 °C for 2 h under permanent nitrogen flow (Air Liquide, 99.99995 %, Algés, Portugal). The characterization of the prepared composite electrocatalysts, Mo_2_C/CNT and Mo_2_C/CXG, by X-ray diffraction analysis (XRD) (Philips 1050 Bruker D8 Advance, Bruker, Billerica, MA, USA), scanning electron microscopy (SEM) (JEOL JSM 7001F, JEOL, Tokyo, Japan), thermogravimetric analysis (TGA) (TA SDT 2960, TA instruments, New Castle, DE, USA), and X-ray photoelectron spectroscopy (XPS) (Kratos AXIS Ultra HSA with VISION software, Kratos Analytical Ltd., Manchester, UK) is presented in detail in a previous paper [24].

Electrocatalysts conductivity measurements (in the form of pressure pelleted powders) were done using Wayne Kerr Universal Bridge B244 (Wayne Kerr, Begnor Regis, SXW, England).

### 2.2. Electrochemical Measurements

All electrochemical measurements were carried out on Gamry PCI4/300 Potentiostat/Galvanostat (Gamry Instruments, Werminster, PA, USA) using a conventional three-electrode system in a single-compartment glass cell of 50 mL volume. The working electrode (Pine Instruments, Durham, NC, USA) was prepared as described below, while a Pt electrode and a saturated calomel electrode (SCE, Hannah Instruments, Woonsocket, RI, USA) served as counter and reference electrode, respectively. All electrode potentials are converted and given relative to the reversible hydrogen electrode (RHE).

Catalytic ink was made by adding 5.0 mg of the electrocatalyst (Mo_2_C/CNT or Mo_2_C/CXG) with 50 μL of Nafion (5 wt.%, Sigma-Aldrich, Taufkirchen, Germany) into 750 μL of ethanol (p.a., Merck, St. Louis, MO, USA) and mixing it ultrasonically for 30 min. The working electrode (Mo_2_C/CNT or Mo_2_C/CXG) was prepared by pipetting 20 μL of the corresponding catalytic ink onto a polished glassy carbon electrode (GCE, 5 mm diameter) and leaving it to dry at room temperature. Current densities were calculated using the geometric surface area of the electrode.

Capacitance behaviour of the two electrocatalysts was studied by recording cyclic voltammograms (CV) in 6 M NaOH (Sigma-Aldrich, Taufkirchen, Germany) and 6 M KOH (Sigma-Aldrich, Taufkirchen, Germany) at different scan rates.

The electrodes were tested for the ORR in 0.1 M NaOH aqueous solutions at room temperature. cyclic voltammetry (CV) and linear scan voltammetry (LSV) measurements were done from 0.2 to 1 V vs. RHE at a scan rate of 10 mV s^−1^. For rotating disk electrode (RDE) measurements, the rotation speed (*ω*) of the electrode was adjusted using a Pine rotator.

## 3. Results

### 3.1. Characterisation of the Electrocatalysts

The activity of two samples towards HER has been tested in the authors’ previous work, along with their characterisation by XRD, SEM, TGA, and XPS [24,41]. XRD analysis confirmed the formation of α-Mo_2_C onto CNTs and CXG and enabled the determination of crystal structure and average crystal size. XRD peaks were assigned to the orthorhombic structure of α-Mo_2_C (ICSD card #1326) and crystallite sizes of 22.3 and 28.6 nm were evaluated for Mo_2_C/CNT and Mo_2_C/CXG, respectively. It should be mentioned that a previous study of ORR at different phases of molybdenum carbide supported on carbon in acidic media have shown that their activity towards ORR is strongly influenced by the carbides’ structure so that α-Mo_2_C/C shows higher activity compared to δ-MoC/C, most likely due to the stronger affinity of the former for O_2_ adsorption [42]. This was further confirmed by the theoretical calculations of O_2_ adsorption energy on the α-Mo_2_C [43] and on the δ-MoC [44]. Thus, O_2_ is predicted to spontaneously dissociate upon adsorption on α-Mo_2_C. 

TGA with linearly rising temperature in air atmosphere was used to determine the actual ratio of Mo_2_C against CNT or CXG, based on the fact that the final product of the analysis is pure MoO_3_. TGA data showed a similar amount of Mo_2_C in both electrocatalysts. For CNT support, 28.5 wt.% of Mo_2_C was incorporated into Mo_2_C/CNT. If CXG is used as support, synthesised electrocatalyst contained 25.6 wt.% of Mo_2_C.

**Figure 1:**
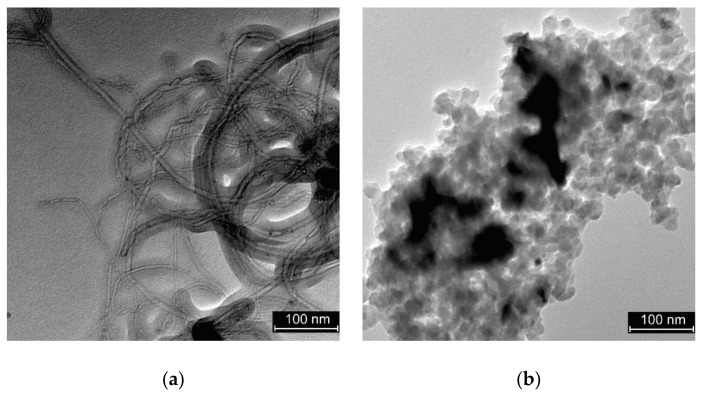
Transmission electron microscopy (TEM) images of: (**a**) molybdenum carbide supported on carbon nanotubes (Mo_2_C/CNT) and (**b**) molybdenum carbide supported on carbon xerogel (Mo_2_C/CXG).

SEM and transmission electron microscopy (TEM) (FEI Tecnai F30, Thermo Fisher, Waltham, MA, USA) analyses revealed significantly different morphologies of the two electrocatalysts. Mo_2_C/CNT showed characteristic tube-like morphology with tube diameters of 10–60 nm and disperse carbide nanoparticles clearly distinguishable in the TEM images (Figure 1a). For the CXG-supported electrocatalyst, particles were found to be agglomerated rather than dispersed (Figure 1b).

Specific surface area and porosity of electrocatalysts were investigated using N_2_-sorption analysis (Quantachrome NOVA 4200e multi-station, Quantachrome Instruments, Boynton Beach, FL, USA). Mo_2_C/CNT was found to be non-porous with a specific surface area of 182 m^2^ g^−1^. For mesoporous Mo_2_C/CXG, an average pore diameter of ca. 18 nm and a specific area of 410 m^2^ g^−1^ were determined.

XPS analysis revealed the presence of Mo in several oxidation states, including Mo 3d doublet located at 228.7 and 231.9 eV, characteristic of Mo_2_C.

### 3.2. Electrical Conductivity Measurements

Non-porous and tube-like structure of a sample enables easier electron transport through the material, so Mo_2_C/CNT showed higher conductivity (10.5 S cm^−1^) compared to Mo_2_C/CXG sample (3.8 S cm^−1^).

### 3.3. The Capacitance Behaviour of Mo_2_C/CXG and Mo_2_C/CNT

**Figure 2:**
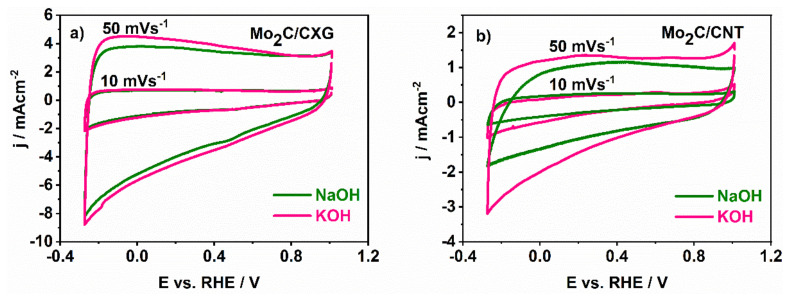
Cyclic voltammograms of (**a**) Mo_2_C/CXG and (**b**) Mo_2_C/CNT in KOH and NaOH aqueous electrolytic solution at scan rates of 10 mV s^−1^ and 50 mV s^−1^.

To characterise the Mo_2_C/CXG and Mo_2_C/CNT interface in an alkaline aqueous solution, cyclic voltammograms of both samples were recorded in KOH and NaOH electrolyte solution (Figure 2). At a low scan rate of 10 mV s^−1^, one can see that the capacitive current does not depend on the type of electrolyte used. Namely, the current response is determined by the sample texture, whereas the K^+^ and Na^+^ ions have enough time to penetrate the pores/spaces equally. At higher rates, the difference between K^+^ and Na^+^ adsorption/desorption processes becomes more pronounced. The ion mobility through the electrolytes turns out to be the determining step at higher scan rates, thus causing the difference in the rate of the electric double layer (EDL) formation. Namely, the higher current response in KOH than in NaOH, measured at 50 mV s^−1^, originates from the faster motion of K^+^ ions through the electrolyte, in other words, the higher transference number of K^+^ vs. Na^+^.

**Figure 3:**
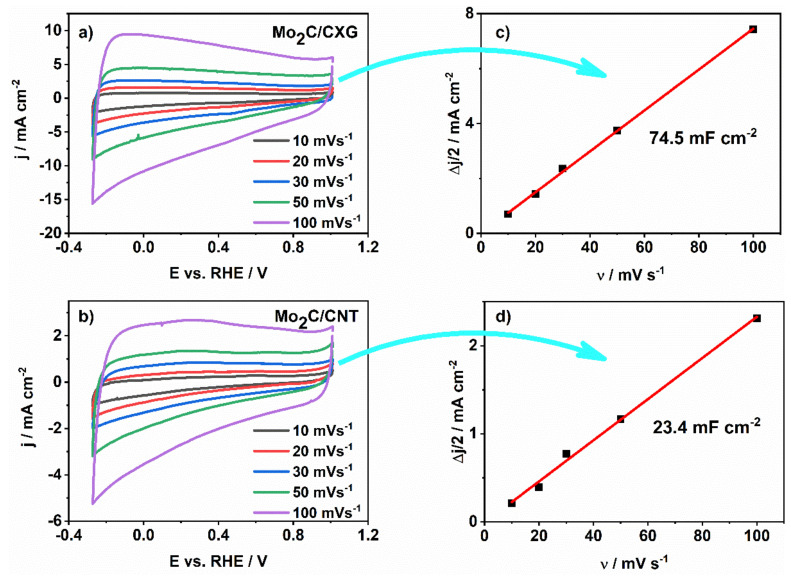
(**a**,**b**) Cyclic voltammograms of Mo_2_C/CXG and Mo_2_C/CNT in 6 M KOH at different scan rates; (**c**,**d**) specific capacitance versus scan rate, calculated from the corresponding cyclic voltammograms for each composite.

The fast EDL formation at the interface of Mo_2_C-based electrodes and KOH is confirmed by the stability of CVs shape upon increasing the scan rate (Figure 3).

**Figure 4:**
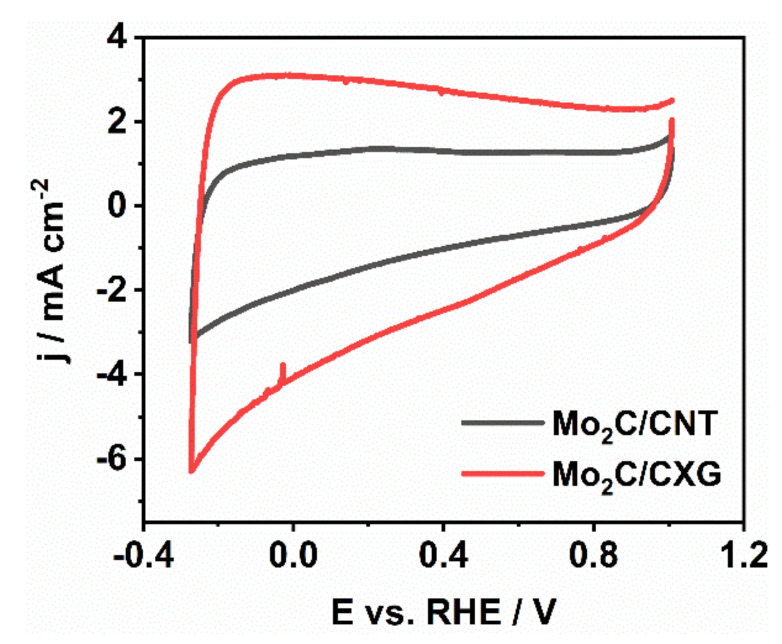
Cyclic voltammograms of Mo_2_C/CXG and Mo_2_C/CNT recorded in 6 M KOH at the scan rate of 50 mV s^−1^.

Double-layer capacity was determined as the slope of ∆*j*/2 vs. *ν* plot (Figure 3c,d), where ∆*j* is the difference of anodic *j*_a_ and cathodic *j*_c_ current densities at a given potential and *ν* is the scan rate. Thus, double-layer capacity was calculated to be 75 mF cm^−2^ and 24 mF cm^−2^ for Mo_2_C/CXG and Mo_2_C/CNT, respectively. This result indicates the higher electrochemically active surface of Mo_2_C/CXG than of Mo_2_C/CNT sample, in agreement with BET (Brunauer–Emmett–Teller) surface area values determined from N_2_-sorption measurements performed in our previous work and evidenced in comparative cyclic voltammograms (Figure 4). The higher current response of Mo_2_C/CXG compared to Mo_2_C/CNT is the consequence of the higher specific surface of CXG-supported Mo_2_C.

To summarize, the background current of the electrical double layer in an alkaline solution is insensitive to the nature of cation at lower scan rates. In contrast, at higher scan rates, it is less pronounced in NaOH than in KOH. For this reason, NaOH was chosen as the conductive electrolytic medium to examine the electrocatalytic activity of samples towards the ORR.

### 3.4. Carbon-Supported Mo_2_C Activity for ORR

**Figure 5:**
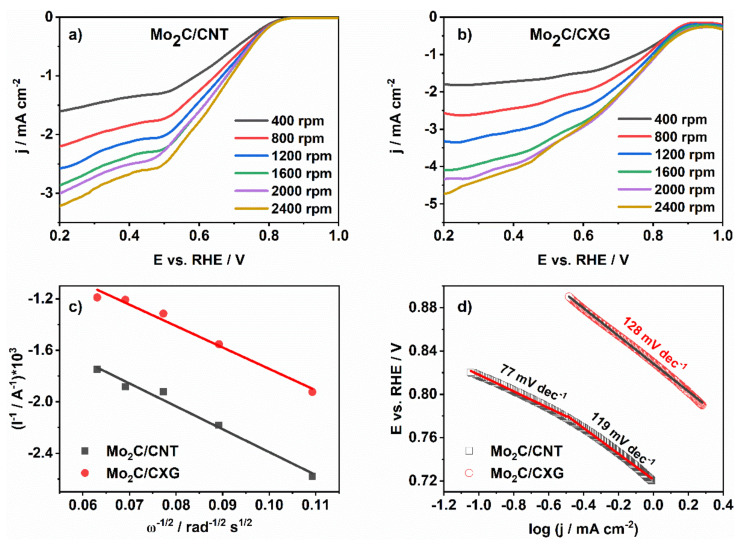
Linear scan voltammograms (LSVs) at different rotation rates for (**a**) Mo_2_C/CXG and (**b**) Mo_2_C/CNT in O_2_-saturated 0.1 M NaOH with (**c**) Koutecky–Levich (K–L) analysis and (**d**) Tafel analysis of oxygen reduction reaction (ORR) at tested samples.

The catalytic activity of carbon-supported Mo_2_C electrocatalysts for ORR was investigated in O_2_-saturated (Messer, 99.9995 vol%, Belgrade, Serbia) 0.1 M NaOH using LSV with a rotating disk electrode (RDE). ORR onset potential was found to be somewhat more positive at Mo_2_C/CXG (0.89 V) compared to Mo_2_C/CNT (0.81 V) (Figure 5) and comparable to the ORR onset potential at similar materials, such as Mo_2_C nanoparticles embedded in nitrogen-doped porous carbon nanofibers (Mo_2_C/NPCNFs) (0.9 V in 0.1 M KOH) [28] or Mo_2_C nanowires (0.87 V in 0.1 M KOH) [45]. Generally, ORR onset potential values for different Mo_2_C-based electrocatalysts were reported to range from 0.75 to 0.91 V [25,26,27]. Furthermore, ORR current densities at Mo_2_C/CXG were found to be higher (−2.9 mA cm^−2^ for Mo_2_C/CXG vs. −1.6 mA cm^−2^ for Mo_2_C/CNT at 0.6 V at 1600 rpm) with ca. 90 mV higher half-wave potential (0.71 V for Mo_2_C/CXG vs. 0.62 V for Mo_2_C/CNT), most likely due to its porosity, higher double-layer capacitance, and larger (active) surface area that accelerate the electron transfer at the electrode/electrolyte interface and enhance its performance towards ORR. For further comparison purposes, ORR current densities at Mo_2_C-based electrocatalysts at 0.6 V at 1600 rpm fall in the range from −2.3 to −4.6 mA cm^−2^ [25,26,27,28].

The reaction kinetics were studied in more detail with the evaluation of key reaction parameters and elucidation of the reaction mechanism. Koutecky–Levich (K–L, Equation (1)) analysis of the background-current corrected LSV curves enabled the determination of the number of electrons exchanged, *n*:

(1) $$\frac{1}{j}=\frac{1}{j_{d}}+\frac{1}{j_{k}}=\frac{1}{0.62nFD^{2/3}\nu^{-1/6}C_{\mathrm{bulk}}\omega^{1/2}}+\frac{1}{j_{k}},$$

where *j*, *j*_k_, and *j*_d_ are measured current density, kinetic current density (free of mass-transfer limitations), and the limiting diffusion current density at the electrode potential *E*, respectively. Additionally, *D* is the O_2_ diffusion coefficient (1.9 × 10^−5^ cm^2^ s^−1^), *ν* is the solution kinematic viscosity (0.01 cm^2^ s^−1^), and *C*_bulk_ is the concentration of dissolved O_2_ (1.2 × 10^−3^ M). As defined, *j*_d_ is determined by the physicochemical properties of the solution and electrode potential *E* in the same manner as *n*. Though K–L analysis is a standard way to determine the number of electrons transferred during the ORR, it should be kept in mind that this analysis is derived for a single one-electron transfer with a preceding chemical reaction of first order and, as such, it might not give absolutely reliable results in case of multi-step reactions involving intermediates [46,47]. Additionally, the K–L analysis might not give reliable results in cases where surfaces may be rough, porous, or both [46] or in case of electrode surface modified with nanoparticles where the real surface area differs from the electrode’s geometric area [47]. Rotating ring disk measurements have been suggested as alternatives, but they still bring some misinterpretation of results due to the theory behind them not being valid for multi-step reactions.

The RDE profiles of two studied carbon-supported Mo_2_C showed increasing current density with increasing rotation rates up to 2400 rpm, indicating a kinetic limitation of the reaction. Constructed K–L plots (Figure 5c) represented straight lines of good linearity, suggesting that ORR is a first-order reaction with respect to O_2_ concentration in the electrolytic solution [27]. For Mo_2_C/CNT and Mo_2_C/CXG, *n* values were determined from the slope of K–L plots to be 2.7 and 3.0 electrons, respectively. Similar behaviour has been reported for the case of unsupported and carbon-supported Mo_2_C nanowires where average *n* during ORR was determined to be 3 in 0.7–0.4 V potential range and 4 in 0.3–0 V potential range. Values from 2.1 to 3.8 have been reported for different Mo_2_C-based electrocatalysts [25,26,27,28]. 

Next, Tafel analysis was carried out providing insight into the mechanism of oxygen adsorption on the surface of two studied samples. ORR Tafel slopes, *b*, were determined from the mass transfer-corrected *j* vs. *E* curves at 1600 rpm, Figure 5d. It has been pointed out that choosing a suitable current range relative to the value of the limiting steady-state current for Tafel analysis might provide more reliable results than choosing a suitable potential range [48]. The suitable current typically lies in the 10–80% range of the limiting current (upon mass-transfer correction). Still, the authors opted for defining a potential range for Tafel analysis as well-defined limiting current densities were not reached.

*b* values for the ORR at Mo_2_C/CNT were evaluated to be 77 mV dec^−1^ (in the low overpotential region where the ORR rate is governed by the rate of surface reaction on the electrocatalyst) and 119 mV dec^−1^. For Mo_2_C/CXG, only one Tafel slope was observed with a value of 128 mV dec^−1^. Tafel slope for ORR at Mo_2_C nanowires was reported to be 65 mV dec^−1^ in low overpotential region (0.87–0.83 V), close to the value for a Pt/C catalyst (64 mV dec^−1^) [45], 60.2 mV dec^−1^ at Mo_2_C/NPCNFs, while Tafel slope of ORR at Mo_2_C NPs embedded in Fe-N-doped carbon nanolayers (Mo_2_C@NC-Fe) was as low as 46 mV dec^−1^ [49].

## 4. Discussion

ORR can occur through two- or four-electron mechanism. The four-electron mechanism implies a direct reduction of molecular oxygen to water by the exchange of four electrons. This is the preferred pathway for oxygen reduction because the alternative two-electron mechanism implies a reduction of molecular oxygen to water via an intermediate step in which peroxide ions are formed. Peroxide can then be reduced to water or can segregate from the electrode and not be involved in the subsequent reaction. ORR on almost all catalysts proceeds by a combination of these two mechanisms where the four-electron mechanism is only dominant in the activation-controlled potential range. One of the most investigated catalysts on which ORR is dominantly occurring through a four-electron mechanism is Pt and Pt-based catalysts; however, even on them, ORR is not occurring by this mechanism in the whole ORR potential range. The number of exchanged electron values obtained herein indicated that ORR at Mo_2_C/CNT and Mo_2_C/CXG is under mixed control where both of the above-mentioned mechanisms are involved, as supported by the appearance of two reduction waves. Previous studies on other Mo_2_C-based electrocatalysts indicated that the ORR at these electrocatalysts also proceeds by a combination of two-electron and four-electron mechanisms or by a two-step two-electron mechanism involving the generation of HO_2_^−^ intermediate [44]. 

Tafel slope values of 119 and 128 mV dec^−1^ determined for Mo_2_C/CNT (high overpotential region) and Mo_2_C/CXG suggest that, though O_2_ adsorbs dissociatively on the Mo_2_C surface at room temperature [42,50], in an aqueous solution, O_2_ and H_2_O adsorption are competing so that O_2_ adsorption might become the rate-determining step [42].

Supporting Mo_2_C nanoparticles on carbon materials, commercial CNTs and prepared CXG, resulted in their good catalytic performance towards ORR for electrochemical energy conversion and storage applications. Their activity is attributed to the synergic effect between carbide species and carbon support. The nature of the catalytic sites (Mo_2_C species, molybdenum oxides, or molybdenum oxycarbide species) for the ORR is still under discussion. Namely, it has been shown that the surface of Mo_2_C particles is contaminated with molybdenum oxides (MoO_3_ and MoO_2_), but their concentration notably decreases upon short activation by galvanostatic electrolysis. The activity of Mo_2_C/CXG was observed to be somewhat higher due to its porosity and large active surface area that accelerate the interfacial electrochemical reaction. In comparison with the available data for other Mo_2_C-based electrocatalysts reported in the literature so far (Table 1), Mo_2_C/CXG shows higher onset potential along with comparable current densities and number of exchange electrons.

Compared to the herein studied catalysts, better results in terms of Tafel slope and current densities at a given potential values display the compositions Mo_2_C/NPCNFs [28] and Mo_2_C@NC-Fe [49]. In the case of the Mo_2_C@NC-Fe catalyst, better performance may be attributed to the promotive effect of ferrous carbide. Considering Mo_2_C/NPCNFs, its synthesis procedure assumed a simultaneous outbreak of molybdenum carbide and carbon support which most likely led to a better contact between them. These facts may serve as directions for future improvement of the herein studied catalysts.

## 5. Conclusions

Within this study, it was shown that the type of cation (K^+^ or Na^+^) in the alkaline electrolytic solution does not affect the rate of the formation of an electrical double layer at low scan rates, since both K^+^ and Na^+^ ions are capable of penetrating the available active sites. However, the different mobility of these cations through the electrolyte is found to be critical for the rate of double-layer formation at higher scan rates. Thus, the capacity of ions that are involved in the EDL formation was higher in KOH than in NaOH, due to the higher mobility of K^+^ ions through the electrolyte. Furthermore, Mo_2_C/CXG shows higher specific capacitance than Mo_2_C/CNT (75 mF cm^−2^ vs. 23 mF cm^−2^), due to the higher specific surface area and porosity.

The catalytic performance of two carbon-supported Mo_2_C in alkaline solution was comparable so that ORR proceeds by a combination of two- and four-electron mechanisms at both electrocatalysts. Mo_2_C/CXG showed somewhat higher activity in terms of the ORR onset potential, half-wave potential, and recorded current densities due to the mentioned higher surface area and a higher number of active sites. Therefore, the use of inexpensive carbides on highly-conductive, high-surface-area carbon nanotubes and carbon xerogel support materials points out a new direction of electrocatalyst performance optimisation for next-generation fuel cells. Further work should include testing of long-term stability of synthesised samples and evaluating the potential application in fuel cells, metal-air batteries, and supercapacitors.

## Figures and Tables

**Table 1:** Comparison of ORR performance of Mo_2_C/CXG and Mo_2_C/CNT with other reported Mo_2_C-based catalysts.

Catalysts	Onset Potential / V	Current Density at 0.6 V ; / mA cm^−2^	Tafel Slope / mV dec^−1^	Electron Transfer Number	Reference
G-Mo_2_C	0.75	−2.3	/	2.1–3.2	[25]
FeMo carbide/NG	0.91	−3.5	/	3.5	[27]
Mo_2_C nanowires	0.87	−2.7	65	3–4	[45]
Hollow Mo_2_C-C microspheres	0.83	−4.2	72.2	3.2–3.6	[26]
Mo_2_C/NPCNFs	0.90	−4.6	60.3	3.8	[28]
Mo_2_C@NC-Fe	/	/	46	3.7	[49]
C(Mo_2_C)	0.84	/	57 (126)	2.8	[51]
Mo-doped MCG	0.76	/	37	2.3	[52]
Mo_2_C/CXG	0.89	−2.9	128	3.0	This work
Mo_2_C/CNT	0.81	−1.6	77 (119)	2.7	This work

G-Mo_2_C—graphite carbon-supported Mo_2_C; NG—nitrogen-doped graphene; NPCNFs—nanoparticles embedded nitrogen-doped porous carbon nanofibers; NC-Fe—Fe-N-doped carbon nanolayers; C(Mo_2_C)—micromesoporous molybdenum carbide-derived carbon powder; MCG—mesoporous carbon/graphene composite.
